# Supplementary material for: Delays in seeking healthcare and its determinants among malaria patients in Ethiopia: A systematic review and meta-analysis
Source: PLoS One. 2025 Apr 8;20(4):e0320149. doi: 10.1371/journal.pone.0320149 (PMC11977998; doi:10.1371/journal.pone.0320149)
Supplement: S2 File — (DOC) [file pone.0320149.s002.docx]

**JBI Critical Appraisal for cross-sectional studies**

| **Author** | **Were the criteria for inclusion in the sample clearly defined?** | **Were the study subjects**  **and the setting described**  **in detail?** | **Was the exposure**  **measured in a valid and reliable way?** | **Were objective, standard criteria used for measurement of the condition?** | **Were confounding factors**  **identified?** | **Were strategies to deal with confounding factors stated?** | **Were the outcomes**  **measured in a valid and reliable way?** | **Was appropriate statistical**  **analysis used?** | **Total score out of 8** |
| --- | --- | --- | --- | --- | --- | --- | --- | --- | --- |
| Workineh and Mekonnen | Yes | Yes | Yes | Yes | no | yes | yes | yes | 7 |
| Dida, Darega et al. | Yes | Yes | No | Yes | yes | yes | yes | yes | 7 |
| Mitiku and Assefa | Yes | Yes | No | Yes | no | yes | yes | yes | 6 |
| Deressa, Ali et al. | Yes | No | No | Yes | yes | yes | yes | yes | 7 |
| Deressa | Yes | Yes | No | Yes | no | yes | yes | yes | 6 |
| Deressa, Chibsa et al. | Yes | Yes | No | Yes | no | yes | yes | yes | 7 |
| \| Birhanu, Abebe et al. \| \| --- \| | Yes | Yes | Yes | Yes | yes | yes | yes | yes | 8 |
| \| Belay, Gelana et al. \| \| --- \| | Yes | No | Yes | yes | yes | yes | yes | yes | 7 |
| \| Dejazach, Alemu et al. \| \| --- \| | Yes | Yes | No | yes | yes | yes | yes | yes | 7 |

Reviewer Moges Tadesse Date June 15, 2024.

**JBI Critical Appraisal for case control studies**

Reviewer Moges Tadesse Date June 15, 2024.

| **Author** | **Were the groups**  **comparable other than the**  **presence of disease in**  **cases or the absence of**  **disease in controls?** | **Were cases and controls**  **matched appropriately?** | **Were the same criteria**  **used for identification of**  **cases and controls?** | **Was exposure measured in**  **a standard, valid, and**  **reliable way?** | **Was exposure measured in**  **the same way for cases**  **and controls?** | **Were confounding factors**  **identified?** | **Were strategies to deal**  **with confounding factors**  **stated?** | **Were outcomes assessed**  **in a standard, valid, and**  **reliable way for cases and**  **controls?** | **Was the exposure period**  **of interest long enough to**  **be meaningful?** | **Was appropriate statistical**  **analysis used?** | **Total score out of 10** |
| --- | --- | --- | --- | --- | --- | --- | --- | --- | --- | --- | --- |
| Goshu and Tafasa | Yes | yes | Yes | Yes | yes | no | yes | yes | no | Yes | 8 |
| Shiferaw, Geremew et al. | Yes | yes | Yes | Yes | yes | no | yes | yes | yes | Yes | 9 |
| Getahun, Deribe et al. | Yes | yes | Yes | Yes | yes | no | yes | yes | no | Yes | 9 |
| Tiruneh, Gebrege et al. | Yes | yes | Yes | Yes | yes | no | yes | yes | no | Yes | 8 |
| Shumerga, Hebo et al. | Yes | yes | Yes | Yes | yes | no | yes | no | no | Yes | 7 |
| Turuse, Gelaye et al. | Yes | no | Yes | Yes | yes | no | yes | yes | no | Yes | 7 |
| Regassa, Taffere et al. | Yes | yes | Yes | Yes | yes | no | yes | yes | no | Yes | 8 |
| Tesfahunegn, Zenebe et al. | Yes | No | Yes | Yes | yes | no | yes | yes | no | Yes | 7 |
| Alga, Wasihun et al. | Yes | yes | No | Yes | yes | no | yes | yes | no | Yes | 7 |

**JBI Critical Appraisal for cross-sectional studies**

Reviewer Muluken Chanie Date June 15, 2024.

| **Author** | **Were the criteria for inclusion in the sample clearly defined?** | **Were the study subjects**  **and the setting described**  **in detail?** | **Was the exposure**  **measured in a valid and reliable way?** | **Were objective, standard criteria used for measurement of the condition?** | **Were confounding factors**  **identified?** | **Were strategies to deal with confounding factors stated?** | **Were the outcomes**  **measured in a valid and reliable way?** | **Was appropriate statistical**  **analysis used?** | **Total score out of 8** |
| --- | --- | --- | --- | --- | --- | --- | --- | --- | --- |
| Workineh and Mekonnen | Yes | yes | Yes | Yes | yes | yes | yes | yes | 8 |
| Dida, Darega et al. | Yes | yes | No | Yes | yes | yes | yes | yes | 7 |
| Mitiku and Assefa | Yes | yes | No | Yes | no | yes | yes | yes | 6 |
| Deressa, Ali et al. | Yes | no | No | Yes | yes | yes | yes | yes | 7 |
| Deressa | Yes | yes | No | Yes | no | yes | yes | yes | 6 |
| Deressa, Chibsa et al. | Yes | yes | No | Yes | no | yes | yes | yes | 7 |
| \| Birhanu, Abebe et al. \| \| --- \| | no | yes | Yes | Yes | yes | yes | yes | yes | 7 |
| \| Belay, Gelana et al. \| \| --- \| | no | yes | Yes | Yes | yes | yes | no | yes | 6 |
| \| Dejazach, Alemu et al. \| \| --- \| | Yes | yes | Yes | Yes | yes | yes | yes | yes | 8 |

**JBI Critical Appraisal for case control studies**

Reviewer Muluken Chanie Date June 15, 2024.

| **Author** | **Were the criteria for inclusion in the sample clearly defined?** | **Were the study subjects**  **and the setting described**  **in detail?** | **Was the exposure**  **measured in a valid and reliable way?** | **Were objective, standard criteria used for measurement of the condition?** | **Were confounding factors**  **identified?** | **Were strategies to deal with confounding factors stated?** | **Were the outcomes**  **measured in a valid and reliable way?** | **Was appropriate statistical**  **analysis used?** | **Total score out of 8** |
| --- | --- | --- | --- | --- | --- | --- | --- | --- | --- |
| Workineh and Mekonnen | Yes | yes | Yes | Yes | yes | yes | yes | yes | 8 |
| Dida, Darega et al. | Yes | yes | No | Yes | yes | yes | yes | yes | 7 |
| Mitiku and Assefa | Yes | yes | No | Yes | no | yes | yes | yes | 6 |
| Deressa, Ali et al. | Yes | no | No | Yes | yes | yes | yes | yes | 7 |
| Deressa | Yes | yes | No | Yes | no | yes | yes | yes | 6 |
| Deressa, Chibsa et al. | Yes | yes | No | Yes | no | yes | yes | yes | 7 |
| \| Birhanu, Abebe et al. \| \| --- \| | no | yes | Yes | Yes | yes | yes | yes | yes | 7 |
| \| Belay, Gelana et al. \| \| --- \| | no | yes | Yes | Yes | yes | yes | no | yes | 6 |
| \| Dejazach, Alemu et al. \| \| --- \| | Yes | yes | Yes | Yes | yes | yes | yes | yes | 8 |

**JBI Critical Appraisal for cross-sectional studies**

| **Author** | **Were the criteria for inclusion in the sample clearly defined?** | **Were the study subjects**  **and the setting described**  **in detail?** | **Was the exposure**  **measured in a valid and reliable way?** | **Were objective, standard criteria used for measurement of the condition?** | **Were confounding factors**  **identified?** | **Were strategies to deal with confounding factors stated?** | **Were the outcomes**  **measured in a valid and reliable way?** | **Was appropriate statistical**  **analysis used?** | **Total score out of 8** |
| --- | --- | --- | --- | --- | --- | --- | --- | --- | --- |
| Workineh and Mekonnen | Yes | yes | Yes | Yes | no | yes | yes | yes | 7 |
| Dida, Darega et al. | Yes | yes | No | Yes | yes | yes | yes | yes | 7 |
| Mitiku and Assefa | Yes | no | No | Yes | no | yes | yes | yes | 5 |
| Deressa, Ali et al. | Yes | no | No | Yes | yes | yes | yes | yes | 7 |
| Deressa | Yes | yes | No | Yes | no | yes | yes | yes | 6 |
| Deressa, Chibsa et al. | Yes | yes | No | Yes | no | yes | yes | yes | 7 |
| \| Birhanu, Abebe et al. \| \| --- \| | Yes | yes | Yes | no | yes | yes | yes | yes | 7 |
| \| Belay, Gelana et al. \| \| --- \| | Yes | yes | Yes | yes | yes | yes | yes | yes | 8 |
| \| Dejazach, Alemu et al. \| \| --- \| | Yes | yes | Yes | yes | yes | yes | no | yes | 7 |

Reviewer Kaleab Tesfaye Date June 15, 2024.

**JBI Critical Appraisal for case control studies**

Reviewer Kaleab Tesfaye Date June 15, 2024

| **Author** | **Were the criteria for inclusion in the sample clearly defined?** | **Were the study subjects**  **and the setting described**  **in detail?** | **Was the exposure**  **measured in a valid and reliable way?** | **Were objective, standard criteria used for measurement of the condition?** | **Were confounding factors**  **identified?** | **Were strategies to deal with confounding factors stated?** | **Were the outcomes**  **measured in a valid and reliable way?** | **Was appropriate statistical**  **analysis used?** | **Total score out of 8** |
| --- | --- | --- | --- | --- | --- | --- | --- | --- | --- |
| Workineh and Mekonnen | Yes | yes | Yes | Yes | no | yes | yes | yes | 7 |
| Dida, Darega et al. | Yes | yes | No | Yes | yes | yes | yes | yes | 7 |
| Mitiku and Assefa | Yes | no | No | Yes | no | yes | yes | yes | 5 |
| Deressa, Ali et al. | Yes | no | No | Yes | yes | yes | yes | yes | 7 |
| Deressa | Yes | yes | No | Yes | no | yes | yes | yes | 6 |
| Deressa, Chibsa et al. | Yes | yes | No | Yes | no | yes | yes | yes | 7 |
| \| Birhanu, Abebe et al. \| \| --- \| | Yes | yes | Yes | no | yes | yes | yes | yes | 7 |
| \| Belay, Gelana et al. \| \| --- \| | Yes | yes | Yes | yes | yes | yes | yes | yes | 8 |
| \| Dejazach, Alemu et al. \| \| --- \| | Yes | yes | Yes | yes | yes | yes | no | yes | 7 |
