# Supplementary material for: Delays in seeking healthcare and its determinants among malaria patients in Ethiopia: A systematic review and meta-analysis
Source: PLoS One. 2025 Apr 8;20(4):e0320149. doi: 10.1371/journal.pone.0320149 (PMC11977998; doi:10.1371/journal.pone.0320149)
Supplement: Attachment — Submitted filename: Review-PONE-D-24-28765.pdf [file pone.0320149.s006.pdf]

Review

The manuscript “Delay in healthcare seeking and its determinants among malaria patients in Ethiopia: a systematic review and meta-analysis” in my opinion requires major revision. The manuscript is difficult to read in several areas and some points are unclear due to “word order” and the language used. Throughout the article, English should be proofread. There are, however, some significant comments that need to be addressed.

Abstract

First, the abstract needs some revisions. The methods must be an accurate reflection of what was done as per the Methods section. The results can be summarised.

2. Introduction

The authors should provide references, when factual statements are made, at the end of the sentence rather than at the end of the paragraph. Paragraphing should be improved and single sentence paragraphs should be avoided.

3. Methods

Google Web has been shown to consistently have the lowest quality relevance ratio and contain many duplicate hits when it comes scientific literature searches. I find it surprising the authors used Google Scholar and Google as well. Authors should provide the MESH terms used and indicate whether the same search strategy was used for all the databases. A repetition of part of the exclusion criteria appears under the Data quality control measures. The sub-heading “Study selection” should be part of the Results.

4. Results

In Table 1 authors should indicate the reference numbers of the studies included in their work. Dejazach, Alemu et al. cannot be found under the Reference section. Several sections of the results need to be rewritten for clarity. Figures have not been numbered making identification difficult. No table 3 seen making it impossible to determine why the 7 factors were chosen.

5. Discussion

Discussion can be markedly improved using recent publications. Some factual statements have been made without any supporting citations. Some sections also need to be rewritten for clarity. The limitations should be expanded.

6. Conclusion

Conclusion has repetitions and this should be addressed.

References

Please review your reference list to ensure that it is complete and correct
