# Supplementary material for: Delays in seeking healthcare and its determinants among malaria patients in Ethiopia: A systematic review and meta-analysis
Source: PLoS One. 2025 Apr 8;20(4):e0320149. doi: 10.1371/journal.pone.0320149 (PMC11977998; doi:10.1371/journal.pone.0320149)
Supplement: Attachment — Submitted filename: PONE-D-24-28765_reviewer.pdf [file pone.0320149.s007.pdf]

PLOS ONE

Delay in healthcare seeking and its determinants among malaria patients in Ethiopia : a systematic review and meta-analysis.

--Manuscript Draft--

**Manuscript Number:**	PONE-D-24-28765
**Article Type:**	Research Article
**Full Title:**	Delay in healthcare seeking and its determinants among malaria patients in Ethiopia : a systematic review and meta-analysis.
**Short Title:**	Delay in malaria healthcare seeking in Ethiopia
**Corresponding Author:**	Moges Tadesse Abebe, MSc<br>Debark University<br>debark, Ethiopia ETHIOPIA
**Keywords:**	delay, healthcare seeking, malaria, Ethiopia
**Abstract:**	Abstract<br>Background<br>Delays in healthcare seeking among malaria patients were associated with an increased risk of severe disease and mortality. Identifying the pooled prevalence and determinants of delay in seeking healthcare in Ethiopia helps to reduce morbidity and mortality among malaria patients.<br>Methods<br>Studies were retrieved from PubMed, Cochrane, Scopus, web of science, Google scholar and Google employing a combination of search terms with Boolean operators. STATA software version 17 was used for all statistical analyses. Heterogeneity across studies was assessed using I2 test. A funnel plot and Egger's weighted regression test at a p value < 0.05 were used to assess the presence of publication bias. Subgroup analyses were performed to explore the possible causes of heterogeneity. Sensitivity analysis was performed to judge whether the pooled effect size was influenced by individual study.<br>Results<br>A total of 18 articles with 7371 participants were included this systematic review and meta-analysis. The pooled prevalence of healthcare seeking delay was 67% (95% CI: 51%, 84%). The visual inspection of the funnel plot showed asymmetrical distribution, and the Egger test was also significant (P = 0.002) which showed the presence of publication bias. Patients less than 15 years old (OR: 2.27, 95%CI: 1.34, 3.85), patients who cannot read and write (OR: 3.36, 95%CI: 1.18, 9.69), patients who went to health institution on foot (OR: 2.77, 95%CI: 1.71, 4.49) and by horse(OR: 2.76, 95%CI: 1.57, 4.84), patients who live far from the health institutions (OR: 2.65, 95% CI: 1.37, 5.13), patients who had family history of death (OR: 3.04, 95%CI: 2.14, 4.33), and patients who were not members of community based health insurance (CBHI) (OR: 7.14, 95%CI: 1.09, 46.63) were significant determinants of delay in seeking health care among malaria patients in Ethiopia.<br>Conclusion<br>In this meta-analysis, the magnitude of malaria health care seeking delay in Ethiopia was high. Healthcare seeking delay was affected by age, literacy level, mode of transportation, distance to the health institution, family history of death and CBHI membership.<br>Keywords: delay, healthcare seeking, malaria, Ethiopia.
**Order of Authors:**	Moges Tadesse Abebe, MSc
	Tesfahun Zemene Tafere
	Kaleab Tesfaye Tegegne
	Dessie Alemnew Shiferaw
	Yosef Aragaw Gonete
	Tadele Kassahun Wudu

Powered by Editorial Manager® and ProduXion Manager® from Aries Systems Corporation

	Getnet Azanaw Takele
	Muluken Chanie Agmasie
**Additional Information:**	
**Question**	**Response**
**Financial Disclosure**<br><br>Enter a financial disclosure statement that describes the sources of funding for the work included in this submission. Review the submission guidelines for detailed requirements. View published research articles from *PLOS ONE* for specific examples.<br><br>This statement is required for submission and **will appear in the published article** if the submission is accepted. Please make sure it is accurate.<br><br>**Funded studies**<br>Enter a statement with the following details:<br>• Initials of the authors who received each award<br>• Grant numbers awarded to each author<br>• The full name of each funder<br>• URL of each funder website<br>• Did the sponsors or funders play any role in the study design, data collection and analysis, decision to publish, or preparation of the manuscript?<br><br>Did you receive funding for this work?	The author(s) received no specific funding for this work.
**Competing Interests**<br><br>Use the instructions below to enter a competing interest statement for this submission. On behalf of all authors, disclose any competing interests that could be perceived to bias this work—acknowledging all financial support and any other relevant financial or non-financial competing interests.<br><br>This statement is required for submission and **will appear in the published article** if	I have read the journal's policy and the authors of this manuscript have no competing interests.

the submission is accepted. Please make sure it is accurate and that any funding sources listed in your Funding Information later in the submission form are also declared in your Financial Disclosure statement.<br><br>View published research articles from *PLOS ONE* for specific examples.<br><br>**NO authors have competing interests**<br><br>Enter: *The authors have declared that no competing interests exist.*<br><br>**Authors with competing interests**<br><br>Enter competing interest details beginning with this statement:<br><br>*I have read the journal's policy and the authors of this manuscript have the following competing interests: [insert competing interests here]*<br><br>* typeset	
**Ethics Statement**<br><br>Enter an ethics statement for this submission. This statement is required if the study involved:<br><br>• Human participants<br>• Human specimens or tissue<br>• Vertebrate animals or cephalopods<br>• Vertebrate embryos or tissues<br>• Field research<br><br>Write "N/A" if the submission does not require an ethics statement.<br><br>General guidance is provided below. Consult the submission guidelines for detailed instructions. **Make sure that all information entered here is included in the Methods section of the manuscript.**	no

Powered by Editorial Manager® and ProduXion Manager® from Aries Systems Corporation

**Format for specific study types**<br><br>**Human Subject Research (involving human participants and/or tissue)**<br>• Give the name of the institutional review board or ethics committee that approved the study<br>• Include the approval number and/or a statement indicating approval of this research<br>• Indicate the form of consent obtained (written/oral) or the reason that consent was not obtained (e.g. the data were analyzed anonymously)<br><br>**Animal Research (involving vertebrate animals, embryos or tissues)**<br>• Provide the name of the Institutional Animal Care and Use Committee (IACUC) or other relevant ethics board that reviewed the study protocol, and indicate whether they approved this research or granted a formal waiver of ethical approval<br>• Include an approval number if one was obtained<br>• If the study involved *non-human primates*, add *additional details* about animal welfare and steps taken to ameliorate suffering<br>• If anesthesia, euthanasia, or any kind of animal sacrifice is part of the study, include briefly which substances and/or methods were applied<br><br>**Field Research**<br><br>Include the following details if this study involves the collection of plant, animal, or other materials from a natural setting:<br>• Field permit number<br>• Name of the institution or relevant body that granted permission	
**Data Availability**<br><br>Authors are required to make all data underlying the findings described fully available, without restriction, and from the time of publication. PLOS allows rare exceptions to address legal and ethical concerns. See the PLOS Data Policy and FAQ for detailed information.	Yes - all data are fully available without restriction

A Data Availability Statement describing where the data can be found is required at submission. Your answers to this question constitute the Data Availability Statement and **will be published in the article**, if accepted.<br><br>**Important:** Stating 'data available on request from the author' is not sufficient. If your data are only available upon request, select 'No' for the first question and explain your exceptional situation in the text box.<br><br>Do the authors confirm that all data underlying the findings described in their manuscript are fully available without restriction?	
**Describe where the data may be found in full sentences. If you are copying our sample text, replace any instances of XXX with the appropriate details.**<br><br>• If the data are **held or will be held in a public repository**, include URLs, accession numbers or DOIs. If this information will only be available after acceptance, indicate this by ticking the box below. For example: *All XXX files are available from the XXX database (accession number(s) XXX, XXX.)*.<br>• If the data are all contained **within the manuscript and/or Supporting Information files**, enter the following: *All relevant data are within the manuscript and its Supporting Information files.*<br>• If neither of these applies but you are able to provide **details of access elsewhere**, with or without limitations, please do so. For example:<br><br>*Data cannot be shared publicly because of [XXX]. Data are available from the XXX Institutional Data Access / Ethics Committee (contact via XXX) for researchers who meet the criteria for access to confidential data.*<br><br>*The data underlying the results presented in the study are available from (include the name of the third party*	available with the manuscript

and contact information or URL).<br>• This text is appropriate if the data are owned by a third party and authors do not have permission to share the data.<br><br>* typeset	
Additional data availability information:	

Manuscript
Click here to access/download;Manuscript;Manuscript.doc

Delay in healthcare seeking and its determinants among malaria patients in Ethiopia: a systematic review and meta-analysis.

Moges Tadesse Abebe[1*], Tesfahun Zemene Tafere[2], Kaleab Tesfaye Tegegne[3], Dessie Alemnew Shiferaw[4], Yosef Aragaw Gonete[5], Tadele Kassahun Wudu[6], Getnet Azanaw Takele[7], Muluken Chanie Agmasie[8]

1. Department of Pediatric and Child health Nursing, College of Health Science, Debark University, Debark, Ethiopia.
   Email: moges7045@gmail.com, *corresponding author
2. Department of Health Systems and Policy, Institute of Public Health, College of medicine and Health Sciences, University of Gondar, Gondar, Ethiopia.
   Email: tesfahunzemene4@gmail.com
3. Department of Public health, College of Health Science, Debark University, Debark, Ethiopia.
   Email: kaleabtesfaye35@gmail.com
4. Department of Nursing, College of Health Science, Debark University, Debark, Ethiopia.
   Email: dessiealem23@gmail.com
5. Department of Midwifery, College of Health Science, Debark University, Debark, Ethiopia.
   Email: yosefaragaw19@gmail.com
6. Department of Statistics, College of Natural and computational science, Debark University, Debark, Ethiopia.
   Email: nabutane2017@gmail.com
7. Department of Midwifery, College of Medicine and Health sciences, University of Gondar, Gondar, Ethiopia.
   Email: getnetelbetel@gmail.com
8. Department of Epidemiology and Biostatistics, Institute of Public Health, College of Medicine and Health Sciences, University of Gondar, Gondar, Ethiopia.
   Email: mulukensrc12@gmail.com

Abstract

Background

Delays in healthcare seeking among malaria patients ~~were~~ associated with an increased risk of severe disease and mortality. Identifying the pooled prevalence and determinants of delay in seeking healthcare in Ethiopia helps to reduce morbidity and mortality among malaria patients.

Methods

Studies were retrieved from PubMed, Cochrane, Scopus, web of science, Google scholar and Google employing a combination of search terms with Boolean operators. STATA software version 17 was used for all statistical analyses. Heterogeneity across studies was assessed using I^2 test. A funnel plot and Egger's weighted regression test at a p value < 0.05 were used to assess the presence of publication bias. Subgroup analyses were performed to explore the possible causes of heterogeneity. Sensitivity analysis was performed to judge whether the pooled effect size was influenced by individual study.

Results

A total of 18 articles with 7371 participants were included this systematic review and meta-analysis. The pooled prevalence of healthcare seeking delay was 67% (95% CI: 51%, 84%). The visual inspection of the funnel

plot showed asymmetrical distribution, and the Egger test was also significant (P = 0.002) which showed the presence of publication bias. Patients less than 15 years old (OR: 2.27, 95%CI: 1.34, 3.85), patients who could not ~~cannot~~ read and write (OR: 3.36, 95%CI: 1.18, 9.69), patients who went to health institution on foot (OR: 2.77, 95%CI: 1.71, 4.49) and by horse(OR: 2.76, 95%CI: 1.57, 4.84), patients who live far from the health institutions (OR: 2.65, 95% CI: 1.37, 5.13), patients who had family history of death (OR: 3.04, 95%CI: 2.14, 4.33), and patients who were not members of community based health insurance (CBHI) (OR: 7.14, 95%CI: 1.09, 46.63) were significant determinants of delay in seeking health care among malaria patients in Ethiopia.

Conclusion

In this meta-analysis, the magnitude of malaria health care seeking delay in Ethiopia was high. Healthcare seeking delay was affected by age, literacy level, mode of transportation, distance to the health institution, family history of death and CBHI membership.

Keywords: delay, healthcare seeking, malaria, Ethiopia.

Introduction

Malaria is caused by obligate intracellular protozoa of the genus *Plasmodium*, including Plasmodium *falciparum*, Plasmodium *malariae*, Plasmodium *ovale*, Plasmodium *vivax*, and Plasmodium *knowlesi (1, 2)*. Of the five *Plasmodium* species, *Plasmodium falciparum is* ~~*responsible*~~ known *to* cause severe malaria (1). This parasite is usually transmitted to humans by female *Anopheles* mosquitoes. It can also be transmitted through blood transfusion, contaminated needles, and transplacentally to a fetus. (Refs?) The most characteristic clinical feature of malaria, which is seldom noted with other infectious diseases, is febrile paroxysms alternating with periods of fatigue but otherwise relative wellness. The classic symptoms of the febrile paroxysms of malaria include high fever, rigors, sweating, and headache (2).

Global malaria prevalence increase~~s~~d from 233 million cases in 2019 to 249 million cases in 2022. Of this, 94% cases were from Africa. In 2022, 608,000 people died worldwide, with more than 50% of those deaths in African countries. What remains? And it remains one of the top ten causes of morbidity and mortality worldwide (3, 4).

Approximately 75% of Ethiopia is at risk of malaria infection. Malaria prevalence in Ethiopia varies by geographic district. Depending on the

nature of the epidemic in each district, the prevalence varies from as low as 5% to as high as almost 50% (5-11).

Early diagnosis and treatment of malaria within 24 hours is the first core principle in malaria treatment (1). A meta-analysis study showed that uncomplicated Plasmodium falciparum infection detected after fever for more than 24 hours ~~was~~ might progress~~ed~~ to severe complicated malaria(12). According to the recent Ethiopian and WHO national malaria control programs, all suspected cases of malaria must be detected and treated within 24 hours of the onset of symptoms to decrease the risk of severe disease, disability and death (13, 14). However, studies conducted in several regions of Ethiopia found that ~~a~~ relatively few patients actually seek medical attention within the recommended 24 hours after the onset of symptoms. Results of studies conducted in Ethiopia have also shown that the prevalence of healthcare seeking among malaria patients after 24 hours of symptom onset varies (15-19).

Since 2011, the implementation of Community Health Insurance (CBHI) in Ethiopia is considered as a means of free medical care that can increase health-seeking behavior throughout the community (20).

Among studies conducted in Ethiopia, several factors influenced delays in seeking malaria treatment. These include socio-demographic factors such as

age, gender, marital status, educational status, occupational status and income. Other determinants include death in the family, side effects of antimalarial drugs, previous malaria infection, behavioral factors such as chat chewing and alcohol consumption, distance and transport-related factors, self-medication practices that account for delayed treatment (21-28). There is also inconsistent finding among different studies about the association of these determinants. For example some studies found healthcare delay was occurred when the household head was decided to seek care (29, 30) while the other study found that early healthcare seeking was occurred when the household head decided to seek care (31).

Therefore, this meta-analysis aimed to pool the prevalence and determinants of delay in healthcare seeking among malaria patients in Ethiopia. This was able to pool the inconsistent findings of the prevalence and determinants among primary studies which is the first review in Ethiopia.

Methods

Reporting

This systematic review and meta-analysis adhered to the Preferred Reporting Items for Systematic Reviews and Meta-Analyses (PRISMA 2020) guideline (32) **(S1 File: PRISMA 2020 Checklist).**

Searching

A search of PROSPERO database was conducted and it was not register in it. Online data bases; PubMed, Cochrane library, Scopus, web of science, Google scholar and Google search were undertaken from May 1/2024 to June 15/2024 for published articles. Unpublished studies were searched from the online Universities institutional repositories. These were followed by analysis of the text words contained in the title and abstract, and of the index terms used to describe article. The referenced lists of all identified researches were searched for additional studies. We conducted the literature search using the medical subject headings (MeSH) and text words. The search terms were: “Malaria”, “patient delay”, “treatment seeking delay”, “healthcare seeking delay", and “Ethiopia”. The Boolean operators (AND and OR) were used to combine terms.

Inclusion criteria

All observational studies conducted on healthcare seeking delay, and/or treatment seeking delays about malaria patients in Ethiopia were

considered. It also included studies which measured the outcome of interest on prevalence and determinants of patient delay, and/or treatment seeking delay among malaria patients in Ethiopia. Furthermore, studies that explained a two by two table of frequency of independent variable with a dependent variable to determine factors affecting healthcare seeking delay were included. In addition, the studies that used 24 hours as a cutoff point to determine presence or absence of malaria healthcare seeking delay were included. Published and unpublished studies written in English language conducted any time in Ethiopia were considered.

Exclusion criteria

Studies that used 48 hours or more as a cutoff point to determine healthcare seeking delay were excluded. Studies that did not explain a two by two table of frequency of health care seeking delay with independent variables to determine factors affecting it, studies that were not fully accessed, studies that did not conduct in English language, studies that did not report patient delay, and/or treatment seeking delay among malaria patients were excluded. The 2023 revised Joanna Briggs Institute (JBI) Critical Appraisal instruments for cross-sectional and case control studies were used to evaluate the methodological quality of each article (33, 34). After evaluation of each study against the criteria, studies with a score of below 5 out of 8 criteria of Critical Appraisal instruments for cross-sectional studies and

below 7 out of 10 criteria for case control were excluded. On the other hand, studies with a minimum score of 5 and 7 or above were included for cross-sectional and case control studies respectively.

The outcome of the study

The outcomes of this review were delay in seeking healthcare and its determinants among malaria patients in Ethiopia.

Operational definitions

Delay in seeking healthcare: The time period from onset of malaria symptoms till first visit to health care facility. The patient was said to be delayed if s/he visited a health facility after 24 hours of onset one of the malaria symptoms.

Far distance: if the patient travel >3k.m distance or takes >30 minutes to reach the health facility.

Symptom: any sensation or change in bodily function that is experienced by a patient

Onset of symptom: It is the time or day when the patient first becomes aware of the symptom or symptoms.

Data quality control measures

Joanna Briggs Institute (JBI) critical appraisal checklist for cross-sectional and case control studies was used to assess quality of the included studies (33). Studies with a score of 5 and more out of 8 criteria for cross-sectional

and 7 and more out of 10 criteria for case control were included. The quality of each study was evaluated independently by two authors (M. T, and M. C). Discrepancies between the two were solved by discussion with third independent reviewer (K.T) **(S2 File: JBI Critical Appraisal).**

Data extraction

The standardized excel data extraction spreadsheet was used to extract data **(S3 file: extracted data).** The name of authors, year of study, region, sample size, frequency of delay and odds ratio with CIs for determinant factors were extracted (Table 1). Two independent reviewers (MT and TK) extracted the data and cross-checked their consistency.

Analysis

Statistical analyses were done by using Stata version 17. The heterogeneity test was conducted by using I-Squared (I^2) statistics. The value of I^2 statistics ranges from 0 to 100% and the 25, 50, and 75%, which represent low, medium, and high heterogeneity across the included studies, respectively. The pooled prevalence of healthcare seeking delay among malaria patients was carried out using a random-effects method due to observed heterogeneity. To reduce the potential random variations between studies; the sources of heterogeneity were analyzed using subgroup analysis. Publication bias was checked by visual inspection of the funnel plots and Egger's weighted regression test at a p-value < 0.05. Asymmetry

of funnel plot was also used as an indication of publication bias. Furthermore, trim and fill analysis was conducted for the presence of publication bias during egger's test. A leave-out-one sensitivity analysis was conducted by excluding each study at a time to identify the impact of each study on the overall estimate.

Study selection

In this systematic review and meta-analysis, a total 1171 articles retrieved from online databases. Among them, 929 duplicated records were removed and 108 articles were excluded through screening the title and abstracts. Then, the remaining 134 full articles were assessed for eligibility; among them, 41 articles did not report the outcome of interest, 7 articles had data inconsistency, and 16 articles had not fulfilled other eligibility criteria. As a result, 70 articles were excluded through reading full texts based on a predefined inclusion, and/or exclusion criteria. Then, 18 research articles were included in the final systematic and review meta-analysis (Fig. 1).

Fig.1: PRISMA Flow chart of study selection for systematic review and meta-analysis of delay in healthcare seeking among malaria patients in Ethiopia

Results

Characteristics of the included studies

This systematic review and meta-analysis included 18 articles with a total of 7371 participants. Among them, eight articles were case control designs (21-23, 25, 26, 29-31) and ten articles were cross-sectional designs (15-17, 19, 27, 28, 35-38). All studies were conducted between 2000 and 2022. Of these studies, 7 studies were from the Oromia region, 6 were from the Amhara region, and 1 study were from the Tigray region, 1 were from Southwest Ethiopia region 1 were from the Central Ethiopia region, 1 from Gambela region and 1 from Benshangul Gumz (Table1).

Table 1: Characteristics of the included studies on healthcare seeking delay among malaria patients in Ethiopia.

s.n.	Author	Study year	Region	Study design	Sample size
1	Goshu and Tafasa	2022	Oromia	Case control	322
2	Shiferaw, Geremew et al.	2016	Southwest	Case control	354
3	Getahun, Deribe et al.	2010	Oromia	Case control	310
4	Tiruneh, Gebrege et al.	2014	Amhara	Case control	318
5	Shumerga, Hebo et al.	2017	Gambela	Case control	306
6	Turuse, Gelaye et al.	2013	Central	Case control	302
7	Regassa, Taffere et al.	2017	Amhara	Cross sectional	394
8	Tesfahunegn, Zenebe et al.	2019	Tigray	Cross sectional	322
9	Workineh and Mekonnen	2017	Amhara	Cross sectional	680
10	Alga, Wasihun et al.	2022	Amhara	Case control	322
11	Dida, Darega et al.	2014	Southwest	Cross sectional	297
12	Mitiku and Assefa	2014	Benshangul Gumz	Cross sectional	302
13	Deressa, Ali et al.	2003	Oromia	Cross sectional	481
14	Deressa	2003	Oromia	Cross sectional	1155
15	Deressa, Chibsa et al.	2000	Oromia	Cross sectional	392
16	Birhanu, Abebe et al.	2014	Oromia	Cross sectional	116
17	Belay, Gelana et al.	2018	Amhara	Cross sectional	246
18	Dejazach, Alemu et al.	2020	Amhara	Cross sectional	149

Prevalence of delay

Ten cross-sectional studies with 4815 participants were incorporated into this category of meta-analysis and the pooled effect size of healthcare seeking delay among malaria patients using the fixed effect model showed significant heterogeneity across the studies with I^2 of 99.6% ($P < 0.001$). Hence, we performed the analysis with a random-effect model with 95% CI to regulate for the observed heterogeneity. Therefore, the overall proportion of delay in healthcare seeking for malaria using the random effects model was 0.67 (95% CI: 0.51, 0.84) (Fig. 2).

Fig. 2: Forest plot showing pooled prevalence of delay in seeking healthcare among malaria patients in Ethiopia.

Subgroup analysis

~~We performed a~~ subgroup analysis based on the region and study setting. ~~The results~~ showed ~~show~~ that the Oromia region had the highest proportion of delayed healthcare seeking (OR: 0.84 95%CI: 0.76, 0.92), and the Amhara region had the lowest proportion of delayed healthcare seeking (OR: 0.44, 95%CI: 0.08, 0.81) (Fig.3). **Fig.3: sub-group analysis by region among malaria patients in Ethiopia.** The proportion was also higher among community based cross-sectional studies (OR: 0.76, 95%CI: 0.63, 0.88) than institution based cross-sectional studies (OR: 0.62, 95%CI: 0.33, 0.92) (Fig.4). **Fig.4: sub-group analysis by study setting among malaria patients in Ethiopia.**

Publication bias and heterogeneity

The presence of publication bias was tested by Egger's test and graphically by using a funnel plot. Visual inspection of the funnel plot revealed an asymmetric distribution (Fig.5). **Fig.5: Funnel plot showing publication bias among studies.** Regression-based Egger test for small-study effects showed p-value of 0.002, indicating publication bias between studies. Therefore, we conducted nonparametric trim and fill analysis of publication bias. Hence, two studies were imputed and we found an effect size of 0.62 (95% CI: 0.48, 0.77)

Sensitivity analysis

We executed a leave-one-out meta-analysis by removing each study step by step to evaluate the effect of a single study on the overall effect estimate. The results indicated that the study conducted by Belay, Gelana et al. influenced the pooled effect size (Fig. 6).

Fig. 6: Sensitivity analysis of pooled prevalence for each study being removed one at a time

Determinants of delay in malaria healthcare seeking

Through eleven studies with 3927 participants, more than 30 variables undergone a meta-analysis to identify factors associated with delay in healthcare seeking among malaria patients in Ethiopia. Of these, 7 variables (patients less than 15 years old, patients who went to health institutions on

foot, patients who went to health institutions by horse, patients who live far from the health institutions, patients who had no family history of death and patients who were not member of CBHI) were significantly associated with delay in healthcare seeking among malaria patients (Table 3).

Age was the determinant of delay in seeking health care among malaria patients. Children less than 15 years were 2.27 times more delayed than adults age greater than 29 (OR: 2.27, 95%CI: 1.34, 3.85).

Patient's literacy level was one of the factors associated with delay in healthcare among malaria patients in Ethiopia. Patients who cannot read and write were 3.36 times more delayed than those with secondary and above educational status (OR: 3.36, 95%CI: 1.18, 9.69).

Mode of transportation was the other determinant of seeking healthcare delay. Patients who went to health institutions on foot were 2.77 times delayed than patients who went by car (OR: 2.77, 95%CI: 1.71, 4.49). In addition, patients who were transported by hoarse were 2.76 times delayed than patients who transported by car (OR: 2.76, 95%CI: 1.57, 4.84).

Patients who were far from health institutions were 2.65 times delayed than patients who came from nearer health institutions (OR: 2.65, 95% CI: 1.37, 5.13).

Experience of death among family members of interviewed patients was also determinants of delay. Patients who had no history of family death

were 3.04 times delayed than patients with a family history of death (OR: 3.04, 95%CI: 2.14, 4.33).

Being a member of CBHI was a significant determinant of delay. Patients who were not members of CBHI were 7.14 time more likely to delay than those who were members of CBHI (OR: 7.14, 95%CI: 1.09, 46.63).

Table 2: A meta-analysis result of factors associated with delay in seeking healthcare among malaria patients in Ethiopia.

Determinants (references)	Comparison	# study	Sample size	OR (95% CI)	P-Value	I^2 (%)	Heterogeneity test (p-value)	Model used
Age less than 15 years (25, 29)	Age greater than 29	2	644	2.27 (1.34, 3.85)	0.002	0.00	0.88	Fixed effect
Cannot read and write (21, 25, 27, 31)	Secondary and above	4	1243	3.36 (1.18, 9.61)	0.024	72.6	0.01	Random effect
Patients who went to health institution on foot (25, 36)	Patients who went to health institution by Car	2	716	2.77 (1.71, 4.49)	<0.001	0.00	0.46	Fixed effect
Patients who went to health institution by hoarse (25, 36)	Patients who went to health institution by Car	2	716	2.76 (1.57, 4.84)	<0.001	0.00	0.66	Fixed effect
Far distance (21-23, 27, 30, 36, 37)	Near distance	7	2655	2.65 (1.37, 5.13)	0.004	89	<0.0001	Random effect
Had no history of death (21, 23, 26, 36)	Had history of death	4	1312	3.04 (2.14, 4.33)	<0.001	0.00	0.79	Fixed effect
No member of CBHI (25, 30)	Member of CBHI	2	322	7.14 (1.09, 46.6)	0.04	96.2	<0.001	Random effect

Discussion

Delays in healthcare seeking among malaria patients increases morbidity and mortality both in the community and specifically in children. Early and effective treatment of malaria is therefore a critical element in malaria control programs as it prevents the progression of the disease to a severe one, reduces malaria related morbidity and mortality and reduces the overall parasite reservoir in the community (39, 40).

The meta-analysis results showed that the overall percentage of delayed healthcare seeking among malaria patients in Ethiopia was 67%. As a result, 67% of patients waited longer than the recommended 24 hours after the onset of malaria symptoms before visiting a health facility. This reflects a failure of communities' timely health-seeking behavior to improve as expected, which may slow progress in malaria control programs to meet national and international goals. To prevent malaria-related complications and death, researchers recommended treating malaria as soon as possible within 24 hours of symptom onset (41). This result was supported by studies conducted in Myanmar(42) and Tanzania (43), but it was lower than study conducted in China in which 85.6% where of patients were delayed (41). This discrepancy could be due to socio-economic differences between these countries? counties. Early treatment-seeking behavior is also important for early case detection and management, thus reducing transmission. Therefore,

promotion of health education to raise awareness is very crucial to direct the wider community to seek timely treatments at health facilities earlier upon the occurrence of malaria symptoms.

Among the various variables analyzed: children less than 5 years, patients who cannot read and write, patients who went to health institutions on foot or by horse, patients who live far from health institutions, patients who were not members of CBHI, patients who had no history of family death were factors associated with delay in healthcare seeking.

The meta-analysis results revealed that the odds of healthcare seeking delay among children age less than 15 years were 2.27 times higher than adults whose age was greater than 29 years old. Children may have difficulty understanding and expressing the symptoms and severity of the illness, causing parents to delay seeking medical attention until their children can recognize the illness and become seriously ill. This finding was also supported by a study conducted in Myanmar (42) and Nigeria (44), but it was contradicted by another study conducted in Myanmar where patients more than 30 years were delayed than younger age (45). This may be the reason why malaria morbidity and mortality is higher in children than in adults. Therefore, parents of children, especially in malaria-endemic areas, must have special health education to seek early healthcare for children with symptoms.

Patients who were unable to read and write were 3.36 times delayed than those with secondary and above educational status. This finding was supported by studies conducted in Myanmar (45), Indonesia (46) and Kenya (47). This may be because as the level of education increases, awareness of the signs and symptoms of the disease and methods of prevention increases, leading to early treatment seeking. The relationship between literacy level and early health-seeking may also be because people who are literate are better able to access and understand health education messages from different media and health professionals.

Patients who were not members of CBHI were 7.14 times more likely to delay than those CBHI members. Health insurance membership avoids patients from paying for labs and medications during a visit, which may be a reason for patients to seek healthcare sooner.

Patients who went to health institutions on foot were 2.82 times delayed than those who went by car. In addition, patients who were transported by horse were also 2.76 times delayed than those who were transported by car. This is because patients may spend long time in traveling long distance on foot or by hoarse to the health institution. They may also delay seeking care because they fear walking long distances or want to wait for their recovery to progress.

Patients who lived far from a health facility delayed 2.65 times more than patients who came from near a health facility. This result was supported by studies conducted in Myanmar(42), Kenya (47) and Equatorial Guinea(48). This may be due to the local advantage of being close to medical facilities. In addition, results also indicate that transportation costs contribute to delays in malaria health visits. Access to health facilities near the patients' villages can make them seek early treatment and increase awareness of early diagnosis and seek timely malaria treatment.

Patients without a family history of death had a 3-fold delay compared to patients with a family history of death. This result was supported by studies conducted in China among Care-seeking delay of imported malaria cases to China (49) and Nigeria (44). This may indicate that patients with a family history of death perceive disease risk and severity to a greater degree than patients without a history of death, suggesting that perceptions of disease risk and severity influence treatment-seeking behavior.

Conclusion

In this meta-analysis, the prevalence of healthcare seeking delay among malaria patients in Ethiopia was high. The healthcare seeking delay varies among different study settings and regions of the country. Furthermore, delay was affected by age, level of education, mode of transportation,

distance to the health institution, family history of death and CBHI membership. The overall determinants of healthcare seeking delay in the current meta-analysis were children less than 15 years, patients who cannot read and write, patients who went to health institutions by foot or horse, patients who live far from health institutions, patients who were not members of CBHI and patients who had patients with a family history of death.

Recommendations

We recommend health extension workers, health professionals and other stakeholders to focus on health education and mobilization of the whole communities on early healthcare seeking for malaria focusing on major symptoms. The health education should provide special focus on children of parents and those who cannot read and write. The government should build infrastructure, particularly roads, to lessen the barrier of transportation in accessing healthcare facilities.

Limitations

Patients and parents of children may not be able to clearly state the exact time of symptom onset, as 24 hours was set as the cut-off point, which may have resulted in selection bias in each study.

Supporting information

S1 File: PRISMA 2020 checklist. (DOC)

S2 File: JBI Critical Appraisal. (DOC)

S3 File: extracted data for prevalence. (XLSX)

Acknowledgments

We would like to thank all authors of studies included in this systematic review and meta-analysis.

Author Contributions

Conceptualization: Moges Tadesse Abebe.

Data curation: Moges Tadesse Abebe.

Formal analysis: Moges Tadesse Abebe, Muluken Chanie Agumasie, Kaleab Tesfaye Tegegne, Dessie Alemnew Shiferaw, Yosef Aragaw Gonete, Tadele Kassahun Wudu, Tesfahun Zemene Tafere, Getnet Azanaw Takele

Investigation: Moges Tadesse Abebe.

Methodology: Moges Tadesse Abebe, Muluken Chanie Agumasie.

Project administration: Moges Tadesse Abebe.

Software: Moges Tadesse Abebe, Tadele Kassahun Wudu.

Supervision: Moges Tadesse Abebe, Muluken Chanie Agumasie, Kaleab Tesfaye Tegegne, Dessie Alemnew Shiferaw, Yosef Aragaw Gonete, Tadele Kassahun Wudu, Tesfahun Zemene Tafere, Getnet Azanaw Takele.

Validation: Moges Tadesse Abebe, Muluken Chanie Agumasie, Kaleab Tesfaye Tegegne, Dessie Alemnew Shiferaw, Yosef Aragaw Gonete, Tadele

Kassahun Wudu, Tesfahun Zemene Tafere, Getnet Azanaw Takele.

Visualization: Moges Tadesse Abebe, Muluken Chanie Agumasie, Kaleab Tesfaye Tegegne, Dessie Alemnew Shiferaw, Yosef Aragaw Gonete, Tadele Kassahun Wudu, Tesfahun Zemene Tafere, Getnet Azanaw Takele.

Writing – original draft: Moges Tadesse Abebe, Muluken Chanie Agumasie, Kaleab Tesfaye Tegegne, Dessie Alemnew Shiferaw, Yosef Aragaw Gonete, Tadele Kassahun Wudu, Tesfahun Zemene Tafere, Getnet Azanaw Takele

Writing – review & editing: Moges Tadesse Abebe, Muluken Chanie Agumasie, Kaleab Tesfaye Tegegne, Dessie Alemnew Shiferaw, Yosef Aragaw Gonete, Tadele Kassahun Wudu, Tesfahun Zemene Tafere, Getnet Azanaw Takele.

References

1. Fikadu M, Ashenafi E. Malaria: An Overview. Infection and drug resistance. 2023;16:3339-47.
2. Kliegman R, Stanton B, St Geme J, Schor N. Behrman RE Nelson Textbook of Paediatrics. Vol. 21. Philadelphia, PA, USA: Elsevier; 2019.
3. Venkatesan P. The 2023 WHO World malaria report. The Lancet Microbe. 2024.
4. Tavares J, Cordeiro-da-Silva A, Calderón F. Ending Malaria: Where Are We? : ACS Publications; 2024. p. 1429-30.
5. Yutura G, Massebo F, Eligo N, Kochora A, Wegayehu T. Prevalence of malaria and associated risk factors among household members in South Ethiopia: a multi-site cross-sectional study. Malaria Journal. 2024;23(1):143.
6. Duguma T, Tekalign E, Muleta D, Simieneh A. Malaria prevalence and risk factors among patients visiting Mizan Tepi University Teaching Hospital, Southwest Ethiopia. PloS one. 2022;17(7):e0271771.
7. Tafesse T, Desalegn Re, Dereje A, Tolera C, Desalegn D, Amenu D. Trends of malaria cases (plasmodium species) in Gute Health Center, Wayu Tuka District, East Wollega Zone,(2013–2022): A cross-sectional study. Health Science Reports. 2024;7(5):e2156.
8. Bidu KT, Babure ZK. Prevalence of malaria and associated factors among febrile patients visiting Kalala Health Center in Haro Limmu Woreda, East Wollega Zone, Western Ethiopia, 2016. Epidemiology (Sunnyvale). 2019;9(365):1165-2161.
9. Tesfaye A, Teshome T. Prevalence and associated factors of malaria infection among outpatients visiting Shewa Robit Health Center, northcentral Ethiopia. Journal of Tropical Medicine. 2022;2022(1):1784012.
10. Lakew YY, Fikrie A, Godana SB, Wariyo F, Seyoum W. Magnitude of malaria and associated factors among febrile adults in Siraro District Public Health facilities, West Arsi Zone, Oromia, Ethiopia 2022: a facility-based cross-sectional study. Malaria Journal. 2023;22(1):259.
11. Dobo B, Fekadu A, Birmeka M. Prevalence of, and risk factors for, malaria infection among patients visiting Goljota Health Center, Heben Arsi District, West Arsi Zone, Oromia Regional State, Ethiopia: A retrospective and an institution-based cross-sectional study. Ethiopian Journal of Health Development. 2021;35(1).
12. Mousa A, Al-Taiar A, Anstey NM, Badaut C, Barber BE, Bassat Q, et al. The impact of delayed treatment of uncomplicated P. falciparum malaria on progression to severe malaria: a systematic review and a pooled multicentre individual-patient meta-analysis. PLoS medicine. 2020;17(10):e1003359.
13. Ababa A. National malaria guidelines. 2018.
14. Organization WH. WHO guidelines for malaria, 14 March 2023. World Health Organization, 2023.
15. Mitiku I, Assefa A. Caregivers’ perception of malaria and treatment-seeking behaviour for under five children in Mandura District, West Ethiopia: a cross-sectional study. Malaria journal. 2017;16:1-10.
16. Deressa W, Ali A, Berhane Y. Household and socioeconomic factors associated with childhood febrile illnesses and treatment seeking behaviour in an area of epidemic malaria in rural Ethiopia. Transactions of the Royal Society of Tropical Medicine and Hygiene. 2007;101(9):939-47.
17. Deressa W, Chibsa S, Olana D. Treatment seeking of malaria patients in East Shewa Zone of Oromia, Ethiopia. Ethiopian Journal of Health Development. 2003;17(1):9-15.
18. Zewdie Birhanu ZB, Yemane Ye-ebiyo Yihdego YY-eY, Delenasaw Yewhalaw DY. Caretakers' understanding of malaria, use of insecticide treated net and care seeking-behavior for febrile illness of their children in Ethiopia. 2017.

19. Belay B, Gelana T, Gebresilassie A. Malaria prevalence, knowledge, attitude, and practice among febrile patients attending Chagni health center, Northwest Ethiopia: a cross-sectional study. Tropical Diseases, Travel Medicine and Vaccines. 2021;7:1-10.
20. Alebachew A, Yusuf Y, Mann C, Berman P. Ethiopia's Progress in health financing and the contribution of the 1998 health care and financing strategy in Ethiopia. MA, Addis Ababa: Harvard TH Chan School of Public Health and Breakthrough International Consultancy, PLC. 2015;95.
21. Turuse EA, Gelaye KA, Beyen TK. Determinants of delay in malaria prompt diagnosis and timely treatment among under-five children in Shashogo Woreda, Hadiya Zone, Southern Ethiopia: a case control study. Health. 2014;2014.
22. Shiferaw D, Geremew M, Kumalo A. Determinants of Delay in Malaria Treatment Seeking for Under-Five Children with Malaria Attending Health Centers of Bench-Maji Zone, South-Western Ethiopia Case-Control Study. Methods. 1930;1:2016.
23. Getahun A, Deribe K, Deribew A. Determinants of delay in malaria treatment-seeking behaviour for under-five children in south-west Ethiopia: a case control study. Malaria Journal. 2010;9:1-6.
24. Deressa W, editor Factors affecting malaria diagnosis and treatment delay among children in east Shewa Zone of Oromia Region, Ethiopia. 28th Annual conference, 2016; 2017.
25. Alga A, Wasihun Y, Ayele T, Endawkie A, Feleke SF, Kebede N. Factors influencing delay in malaria treatment seeking at selected public health facilities in South Gonder, Ethiopia. Scientific reports. 2024;14(1):6648.
26. Shumerga AT, Hebo HJ, Gebrehiwot TT, Gebre MN. Determinants of Delay in Seeking Malaria Treatment for Under-Five Children at Gambella Town, Southwest Ethiopia: A Case-Control Study. Journal of Tropical Medicine. 2020;2020(1):2310971.
27. Dida N, Darega B, Abebe A. Treatment-seeking behavior and associated factors among malaria suspected patients in Bale Zone, Southeast Ethiopia: institution-based cross-sectional study. J Fam Med. 2015;2(1):5.
28. Deressa W. Treatment-seeking behaviour for febrile illness in an area of seasonal malaria transmission in rural Ethiopia. Malaria Journal. 2007;6:1-7.
29. Goshu CJ, Tafasa SM. Determinants of delay in treatment seeking among malaria patients in public health facilities of Dambi Dollo and Sayyo district, Western Ethiopia: A facility based case control study. Open Access Journal of Disease and Global Health. 2023;1(1):6-20.
30. Melkamu Tiruneh MT, Gebremedhin Berhe Gebregergs GBG, Dereje Birhanu DB. Determinants of delay in seeking treatment among malaria patients in Dera district, NorthWest Ethiopia: a case control study. 2018.
31. Tesfahunegn A, Zenebe D, Addisu A. Determinants of malaria treatment delay in northwestern zone of Tigray region, Northern Ethiopia, 2018. Malaria journal. 2019;18:1-8.
32. Page MJ, McKenzie JE, Bossuyt PM, Boutron I, Hoffmann TC, Mulrow CD, et al. The PRISMA 2020 statement: an updated guideline for reporting systematic reviews. International journal of surgery. 2021;88:105906.
33. Barker TH, Stone JC, Sears K, Klugar M, Leonardi-Bee J, Tufanaru C, et al. Revising the JBI quantitative critical appraisal tools to improve their applicability: an overview of methods and the development process. JBI Evidence Synthesis. 2023;21(3):478-93.
34. Barker TH, Stone JC, Sears K, Klugar M, Tufanaru C, Leonardi-Bee J, et al. The revised JBI critical appraisal tool for the assessment of risk of bias for randomized controlled trials. JBI evidence synthesis. 2023;21(3):494-506.
35. Birhanu Z, Abebe L, Sudhakar M, Dissanayake G, Yihdego YY-e, Alemayehu G, et al. Malaria related perceptions, care seeking after onset of fever and anti-malarial drug use in malaria endemic settings of southwest Ethiopia. PloS one. 2016;11(8):e0160234.

36. Regassa H, Taffere GR, Gebregergs GB. Delay in malaria diagnosis and treatment and its determinants among rural communities of the Oromia special zone, Ethiopia: facility-based cross-sectional study. Journal of Public Health. 2018;26:339-44.
37. Baymot Workineh BW, Fantahun Ayenew Mekonnen FAM. Early treatment-seeking behaviour for malaria in febrile patients in northwest Ethiopia. 2018.
38. Dejazmach Z, Alemu G, Yimer M, Tegegne B, Getaneh A. Prevalence of malaria and associated knowledge, attitude, and practice among suspected patients in Bahir Dar Zuria District, Northwest Ethiopia. BioMed research international. 2021;2021(1):3741413.
39. Organization WH. WHO informal consultation on fever management in peripheral health care settings: a global review of evidence and practice. 2013.
40. Committee WMPA, int Smw. Malaria Policy Advisory Committee to the WHO: conclusions and recommendations of September 2013 meeting. Malaria Journal. 2013;12(1):456.
41. Jia L, Chen X, Feng Z, Tang S, Feng D. Factors affecting delays in seeking treatment among malaria patients during the pre-certification phase in China. Malaria Journal. 2024;23(1):73.
42. Xu J-W, Xu Q-Z, Liu H, Zeng Y-R. Malaria treatment-seeking behaviour and related factors of Wa ethnic minority in Myanmar: a cross-sectional study. Malaria journal. 2012;11:1-7.
43. Kassile T, Lokina R, Mujinja P, Mmbando BP. Determinants of delay in care seeking among children under five with fever in Dodoma region, central Tanzania: a cross-sectional study. Malaria journal. 2014;13:1-10.
44. Chukwuocha UM, Okpanma AC, Nwakwuo GC, Dozie INS. Determinants of delay in seeking malaria treatment for children under-five years in parts of South Eastern Nigeria. Journal of community health. 2014;39:1171-8.
45. Than MM, Min M, Aung PL. The determinants of delayed diagnosis and treatment among malaria patients in Myanmar: a cross-sectional study. The Open Public Health Journal. 2019;12(1).
46. Guntur RD, Kingsley J, Islam FMA. Malaria treatment-seeking behaviour and its associated factors: A cross-sectional study in rural East Nusa Tenggara Province, Indonesia. PloS one. 2022;17(2):e0263178.
47. Sumba PO, Wong SL, Kanzaria HK, Johnson KA, John CC. Malaria treatment-seeking behaviour and recovery from malaria in a highland area of Kenya. Malaria Journal. 2008;7:1-8.
48. Romay-Barja M, Cano J, Ncogo P, Nseng G, Santana-Morales MA, Valladares B, et al. Determinants of delay in malaria care-seeking behaviour for children 15 years and under in Bata district, Equatorial Guinea. Malaria journal. 2016;15:1-8.
49. Lu G, Cao Y, Chen Q, Zhu G, Müller O, Cao J. Care-seeking delay of imported malaria to China: implications for improving post-travel healthcare for migrant workers. Journal of travel medicine. 2022;29(4):taab156.

Click here to access/download;Figure;Fig. 1.pdf

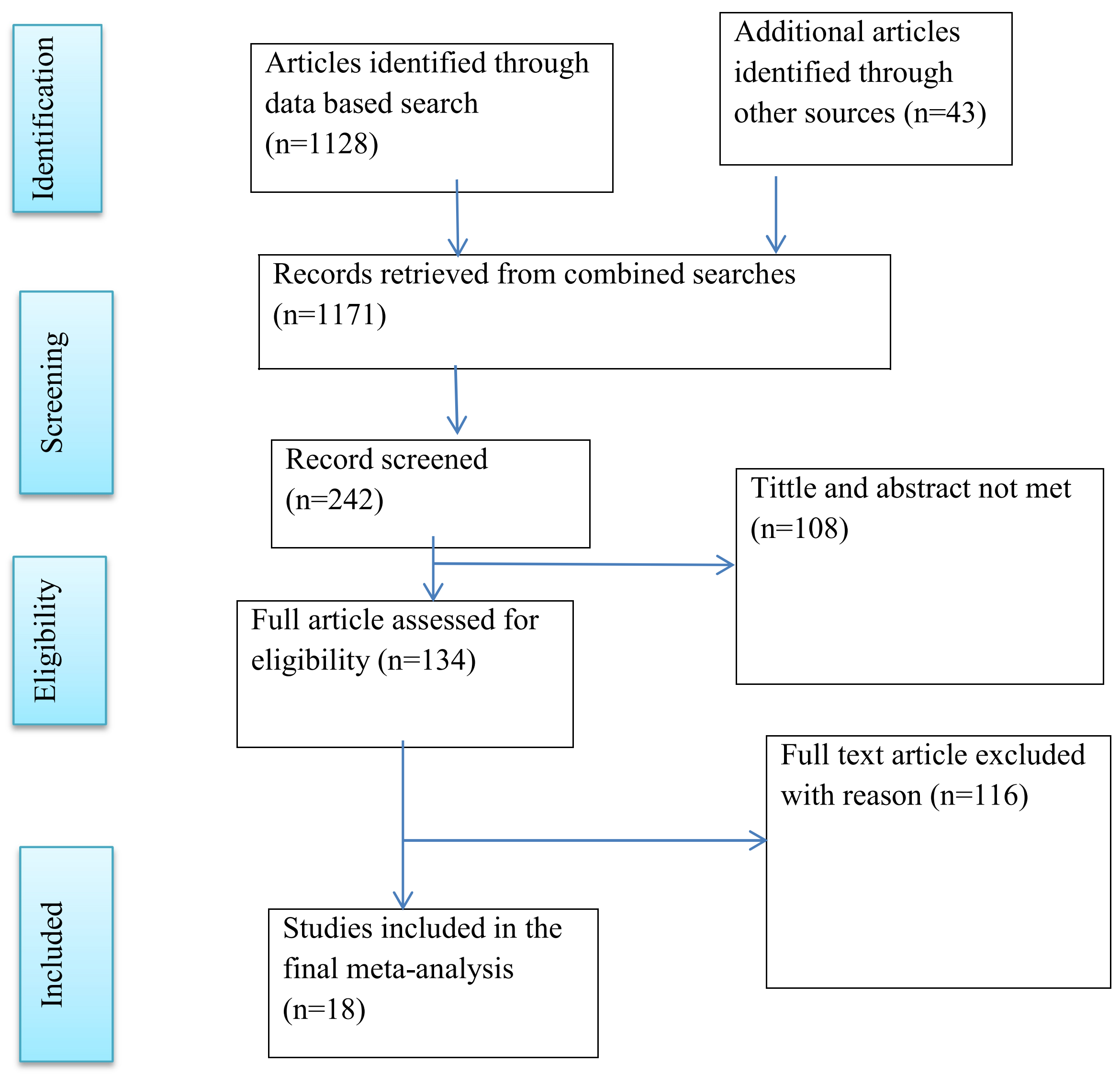

Fig. 1: PRISMA Flow chart of study selection for systematic review and meta-analysis of delay in healthcare seeking among malaria patients in Ethiopia

Click here to access/download;Figure;Fig. 2.jpg

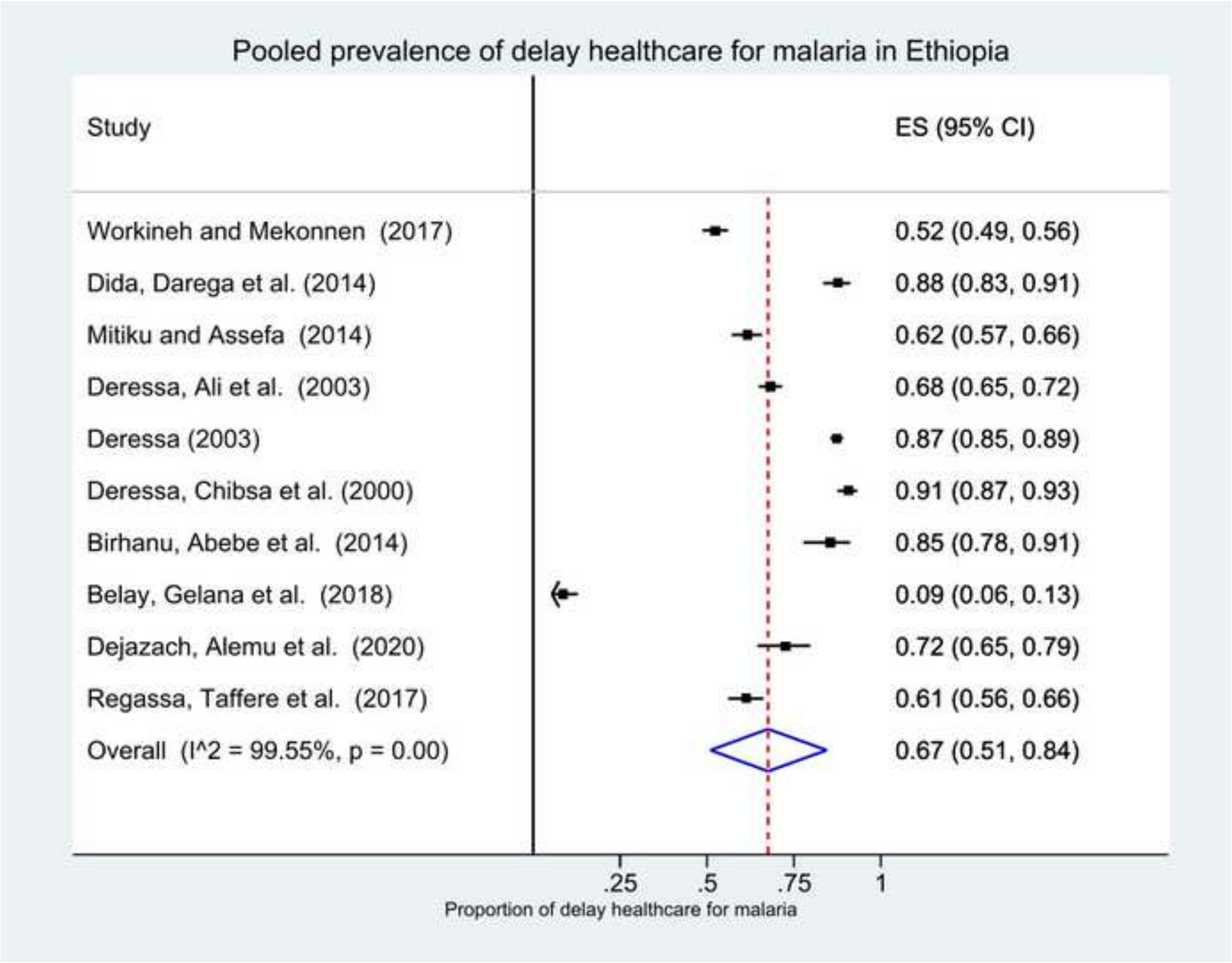

Click here to access/download;Figure;Fig. 3.jpg

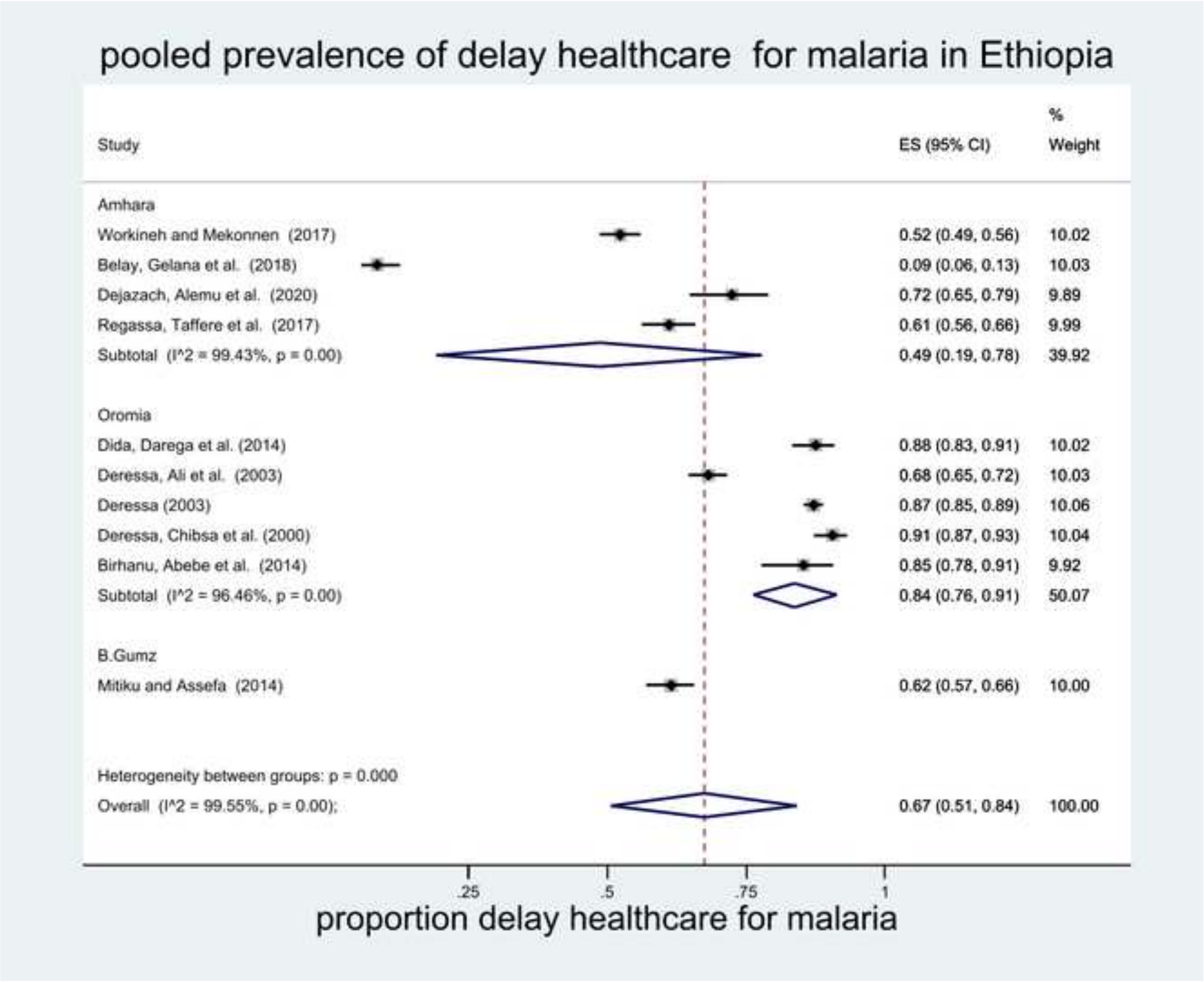

Click here to access/download;Figure;Fig. 4.jpg

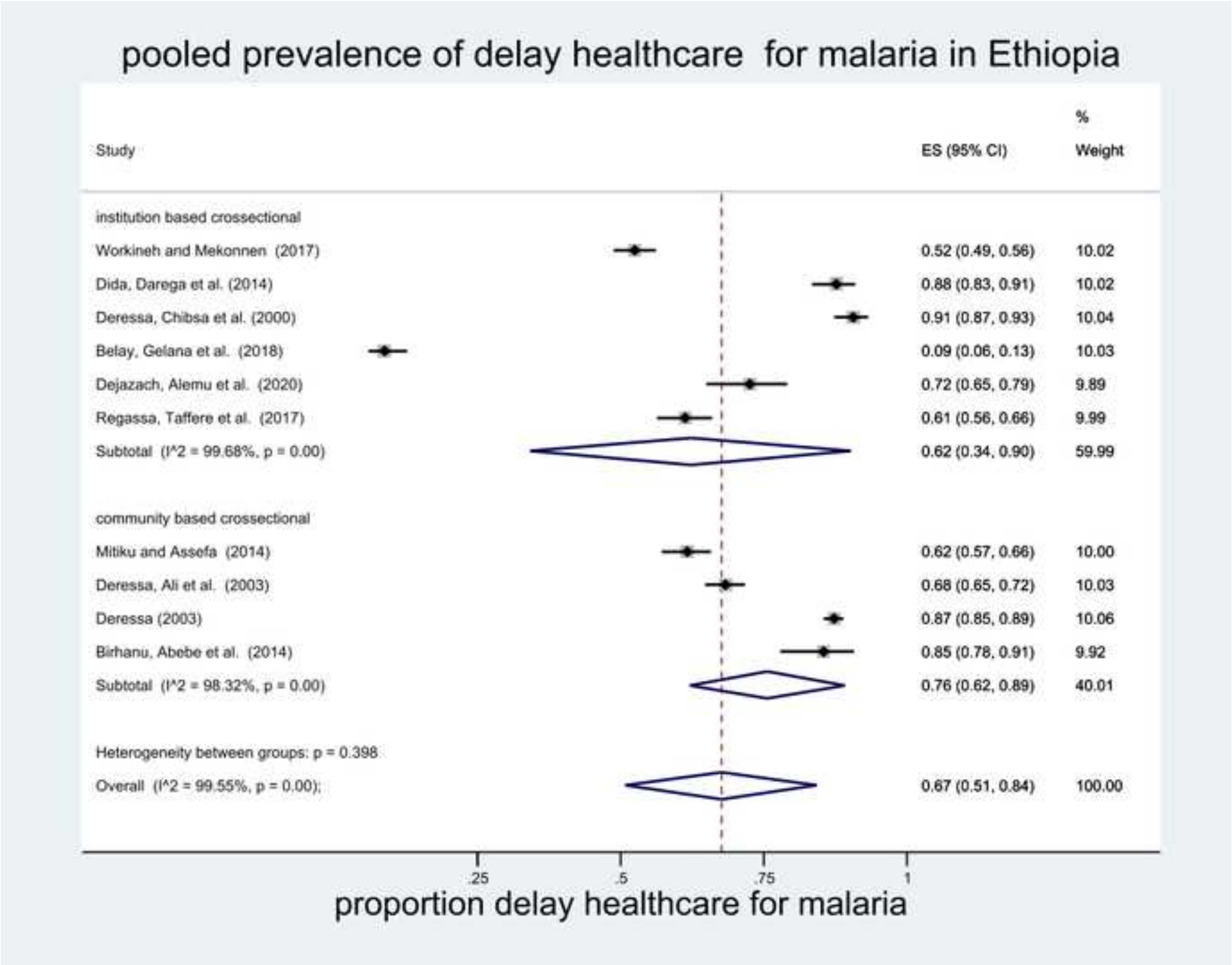

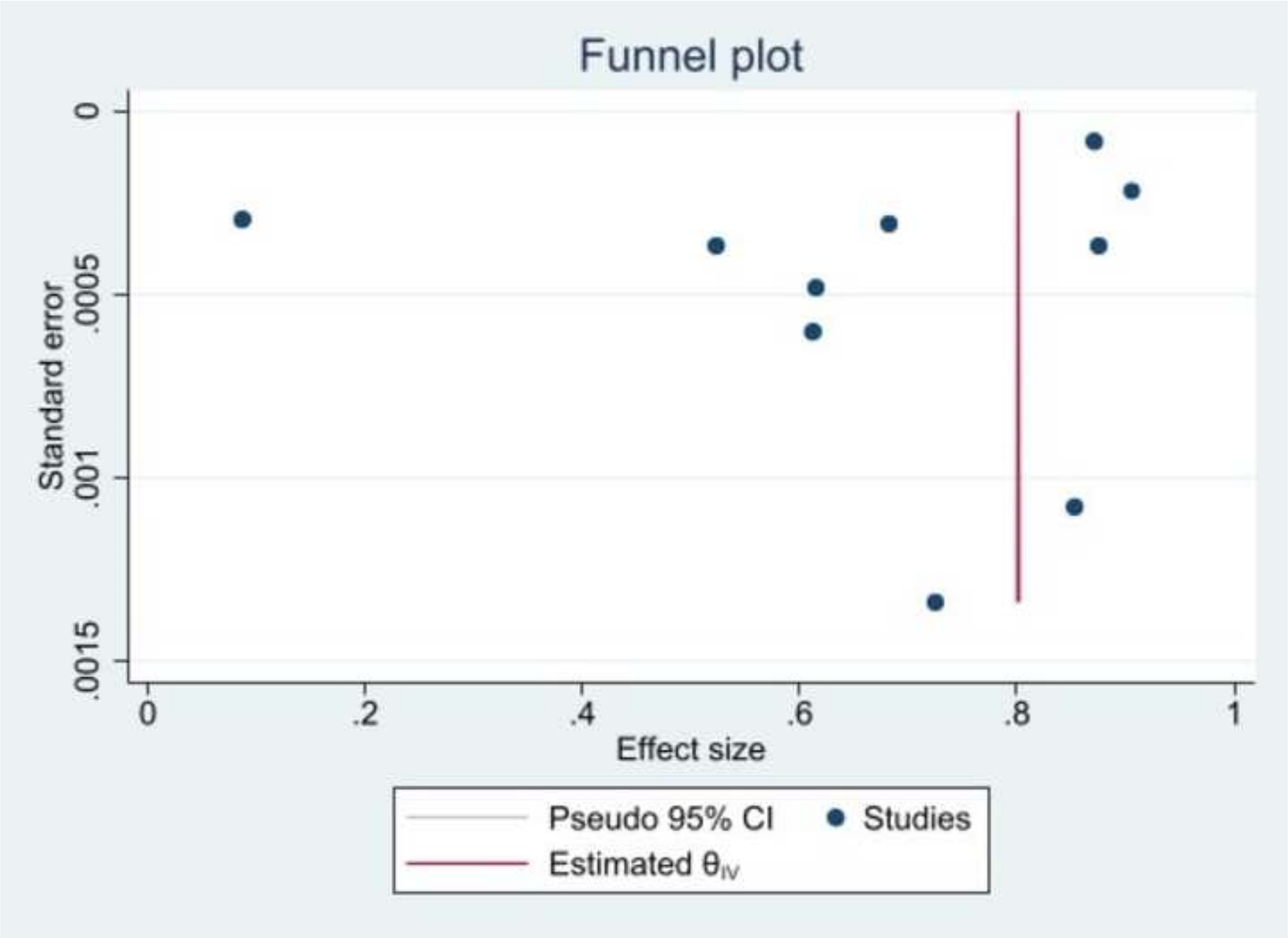
Funnel plot
Standard error
0
.0005
.001
.0015
0
.2
.4
.6
.8
1
Effect size
Pseudo 95% CI
Studies
Estimated θIV

Click here to access/download;Figure;Fig. 6.jpg

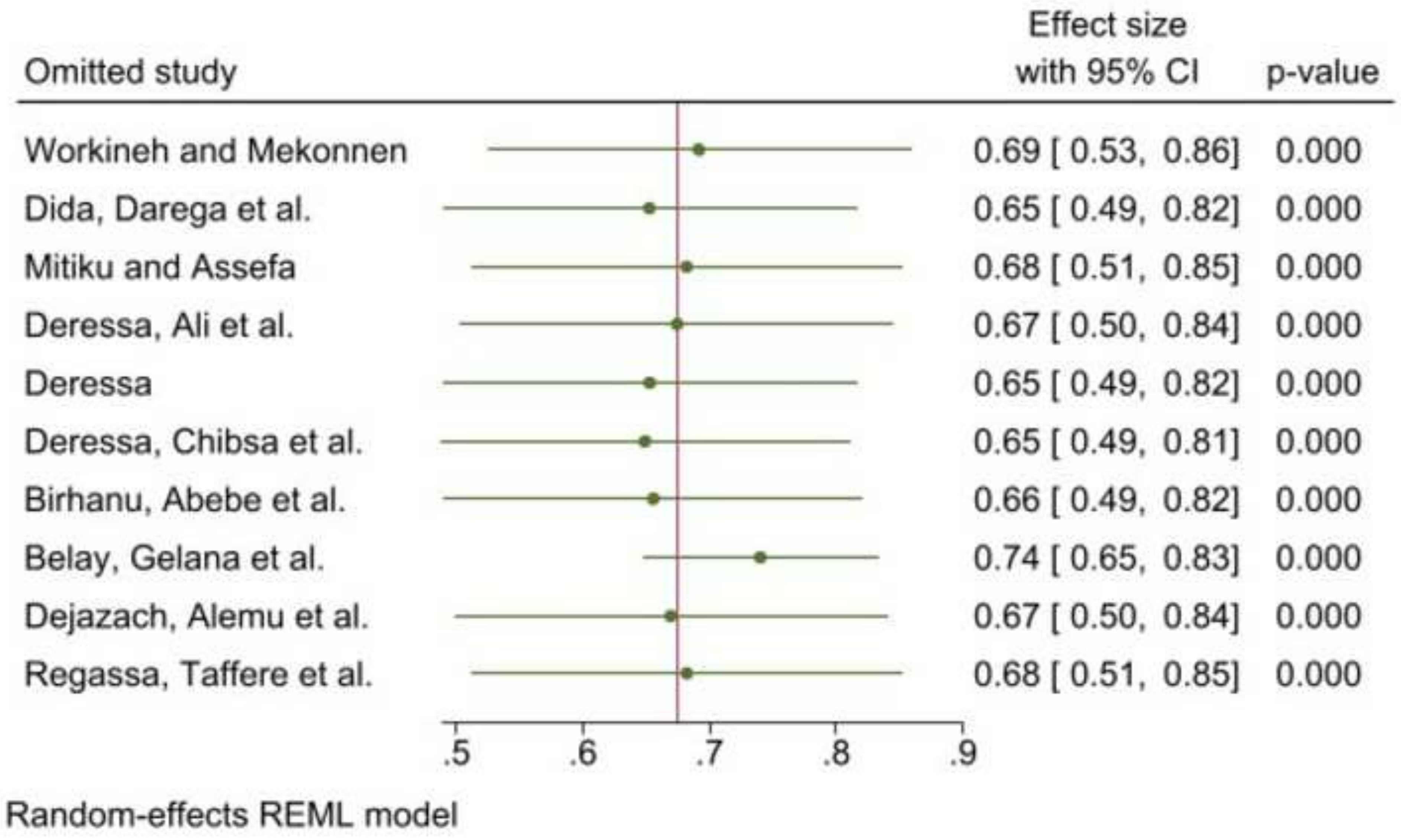

Click here to access/download
Supporting Information
S1 file PRISMA 2020.docx

Click here to access/download
Supporting Information
S2 file JBI critical appraisal.docx

Click here to access/download
Supporting Information
S3 file extracted data p.xls
