# Supplementary material for: Delays in seeking healthcare and its determinants among malaria patients in Ethiopia: A systematic review and meta-analysis
Source: PLoS One. 2025 Apr 8;20(4):e0320149. doi: 10.1371/journal.pone.0320149 (PMC11977998; doi:10.1371/journal.pone.0320149)
Supplement: Attachment — Submitted filename: Abebe et al PLOS One Review Dec 2024.docx [file pone.0320149.s005.docx]

**Review of Abebe et al 2024**

General comments:

- Overall, an important meta-analysis for malaria care in Ethiopia but improvements are required.
- Authors indicate that data is available but not where it is available from. Suggest copying example statement to indicate that all data is available in the manuscript or supplementary material
- Have indicated some instances were English/grammar should be revised through the beginning of the methods but overall, the manuscript would benefit from review for English and grammatical correctness, as well as from copyediting.
- Consistently write healthcare or health care.
- Check Italicizations.
- Check tense used.
- The introduction lists all these determinants for delays in care seeking. I think the rationale for this meta-analysis needs to be more clearly articulated.
- Google (vs Google Scholar) is not usually used as a search database for systematic reviews. What was the justification of including it as one?
- Flow of the discussion could be improved.

Specific comments:

| Abstract | Methods section of abstract suggest consistently capitalization the names of databases searched. |
| --- | --- |
|  | Last sentence of the methods would add “a” before “individual study” |
|  | In the results, would suggest presenting the confidence interval as a range as opposed to using a comma between the start and end of the range. |
|  | With a p-value of 0.002 for the Egger test would this suggest that that it is highly significant not just significant? |
|  | In the last sentence of the results section, you list the determinants of delay in seeking healthcare among malaria patients in Ethiopia. I would suggest omitting patients before each determinant. For instance instead of writing, “patients less than 15 years old” I would write, “being under 15 years old, not being able to read and write, traveling to the health center on foot,…, were significant determinants of delay……” because the patients themselves are not the determinants. |
|  | In the conclusion of the abstract, it summarizes what was already presented in the results. Are there any broader conclusions or suggestions that can be made based on these findings? |
| Introduction | Consistent italicizing of full malaria species name (including the genus) |
|  | Page 4: Suggest changing responsible to cause…. “To responsible for most cases of severe malaria.” Though less common, other species can also cause severe malaria. |
|  | Page 4: “Global malaria prevalence increased.....” Change increases to increased. |
|  | Page 4: Do not need the “And” at the start of the last sentence of the second paragraph. |
|  | Page 4-5: “Depending on the nature of the epidemic in each district..” change to “Depending on transmission dynamics in each district…. |
|  | Page 5: it is unclear what the meta-analysis indicates mentioned in the second sentence of the second paragraph. That is not treated within 24 hours that it would progress to severe malaria? If so, would clarify this. |
|  | Page 5: “According to the recent Ethiopian and WHO national malaria control programs,…” recent what? And the WHO does not have a national program, so according to recent evidence, the WHO and Ethiopian national malaria control program,…..” |
|  | Page 5, paragraph 3: CBHI acronym, missing the B in the full written name? Community-Based Health Insurance? |
|  | Page 6: would explain what “chat” is for those who may not know. |
|  | Page 6: “There is also inconsistent finding among…” 🡪 “There are also inconsistent findings among…” |
|  | Page 6: Last sentence of the first paragraph needs clarifying. It does not make sense. Seems both findings are the same. Tense use is also incorrect. |
| Materials and Methods | Page 7, under searching: in the first sentence, would clarify what was not registered in PROSPERO. |
|  | Page 7: Consistent capitalization of databases searched.  A search was understand taken, not were. |
|  | Page 7: what universities’ institutional repositories? |
|  | Page 8: All inclusion criteria after the first one listed, just appear to be more specifics about the first inclusion criteria. If so, should they be inclusion criteria? They fit better as listed in the exclusion criteria. |
| Results | Page 14: Prevalence of delay: suggest to maybe clarify why those 10 cross-sectional studies were included in the meta-analysis (as opposed those that were not)? |
|  | Page 14: add a comma in the first sentence after meta-analysis |
|  | Page 14: here results are presented from a subgroup analysis. I would suggest adding more detail about this in the methods section. Also is this subgroup analysis methodologically sound since some regions had a lot fewer studies conducted than others? |
|  | Page 15-16: I would suggested in the last sentence of the last paragraph to change it to “Of these patient-related variables, seven of them (being less than 15 years old, xxxxx),… were significantly associated with delay…” The patients themselves are not the variables. |
|  | Page 16: “Age was the determinant of delay in seeking health care among malaria patients.” Here to you mean that age has the most impact? For all the different factors you talk about, I would make a statement as to how their impact relates to one another. |
|  | Page 16: Misspelling of horse in the second paragraph |
| Discussion | Page 18: “This result was supported by studies conducted in Myanmar(42) and Tanzania (43), but it was lower than study conducted in China in which 85.6% where of patients were delayed (41).” Would specify what result you mean here, since the sentence before you are talking about the recommendation. |
|  | Overall, the flow of the discussion is okay, though within some paragraphs the flow is a bit disjointed with findings, other studies, and recommendations not discussed at once and broken apart. |
|  | Suggest having a stronger strength and limitations section/paragraph at the end of the discussion. |
